# Editorial: Aquatic animal health and epidemiology: disease surveillance, prevention and control

**DOI:** 10.3389/fvets.2026.1798114

**Published:** 2026-03-09

**Authors:** Prabhugouda Siriyappagouder, Naveen Kumar B. T., Balbir Bagicha Singh, Krishna Thakur, Azad Ismail Saheb, Beng Chu Kua

**Affiliations:** 1 Faculty of Biosciences and Aquaculture, Nord University, Bodø, Norway; 2 Indian Council of Agricultural Research (ICAR)-Central Marine Fisheries Research Institute, Mandapam Regional Center, Tamil Nadu, India; 3 Guru Angad Dev Veterinary and Animal Sciences University, Ludhiana, India; 4 University of Prince Edward Island, Charlottetown, PE, Canada; 5 Kuwait Institute for Scientific Research, Salmiya, Kuwait; 6 Fisheries Research Institute, Department of Fisheries Malaysia, Batu Maung, Penang, Malaysia

**Keywords:** diagnostics, disease surveillance and prevention, epidemiology, fish health, vaccine

Aquatic animal disease is a major concern and a significant challenge to achieving sustainable aquaculture production and meeting food security goals. Managing aquatic animal health requires multidisciplinary approaches, including, but not limited to, pathology, epidemiology, marine and environmental sciences, and molecular biology. Early diagnostic tools play a key role in identifying pathogens and diseases, while prophylactic strategies such as vaccination, biosecurity measures, and good husbandry practices are vital for effectively controlling disease outbreaks. Our research collection Aquatic Animal Health and Epidemiology: Disease Surveillance, Prevention, and Control includes 10 research and review articles that have advanced our understanding of host-pathogen interactions, pathology, pathogen diversity, and their molecular characterization, as well as diagnostics and predictive surveillance modeling of disease dynamics within aquatic environments.

Environmental stressors are increasingly affecting aquatic animal health, especially in open-sea cages, as climate change alters environmental conditions. A major challenge is maintaining optimal dissolved oxygen levels in cages. Cerqueira et al. developed a predictive model to estimate oxygen concentrations in cages. Factors such as rising water temperatures, algal blooms, eutrophication, and high stocking density reduce oxygen levels, leading to hypoxic conditions. Early detection of these conditions is crucial to reducing fish stress and improving their welfare. To address this, a machine learning method was used to predict hypoxia events in salmon farms.

The emergence of new diseases remains a serious concern due to constantly shifting environmental conditions, interactions, and pathogen exchange between wild and farmed fish populations. Efforts continue to reduce the risk of pathogen transmission between wild and farmed populations, as well as from farms to the environment. Wassmuth et al. explore this issue in their review, focusing on Salmonids and *Tenacibaculum maritimum*. They emphasize the importance of several factors, including host biology, pathogen traits, and environmental factors. The review recommends combining ecological and management approaches to effectively control bacterial infections in salmonid farming.

Microbial communities associated with the host and the surrounding environment are known to modulate the health of many aquatic species, including oysters. Some microbes cause disease, and others are beneficial to the host. Cram et al. characterized variability in microbial community structure across batches of oyster larvae and found that rapid changes in microbial composition can affect larval performance and survival. In addition, microeukaryotes, such as harmful algae, can negatively affect oyster larvae, leading to crashes in oyster larvae batches. Therefore, identifying and characterizing microbial community composition is vital for sustaining the hatchery's health and can be considered a key component of an early warning system addressing not only bacteria but also other biological entities, including parasites.

Suwannarat et al. characterized three pathogenic digenean parasites of marine fish in the Gulf of Thailand using a combination of morphological and molecular analyses. The authors believe this study provides new data on the underexplored helminths of marine fish and suggest that further research is needed to understand the comprehensive patterns of host-parasite interactions and host specificity.

Similarly, viral diseases are among the most critical challenges in fish health due to the lack of effective viral treatments. Although many attempts are made to combat viral infections, such as selective breeding for resistant strains and vaccine development, these efforts still fail to yield effective strategies. Despite endless interest in developing new antiviral therapeutics, Pourhoseini Dehkordi et al. explored the use of interfering RNA (RNAi), particularly shRNA, which targets the non-virion (NV) gene of viral hemorrhagic septicemia virus (VHSV), and it exhibited strong antiviral properties and prevented proliferation of viral hemorrhagic septicemia virus (VHSV). Therefore, gene-silencing technologies are proposed as a possible method to prevent viral infections.

Nonetheless, the significance of vaccination in fish health management cannot be overlooked. Vaccines may vary in effectiveness, but they remain among the most effective preventive strategies for controlling fish diseases in aquaculture. Onireti et al. assessed the safety and effectiveness of an autogenous *Vibrio anguillarum* vaccine in both controlled and field conditions. The vaccine offered long-lasting immunity and protected against the challenge but did not provide cross-protection against other strains of *V. anguillarum*. It provided a level of protection similar to that of a commercial vaccine under field conditions for lumpfish in sea cages.

Vaccines, functional feeds, and biosecurity measures are essential to maintaining fish health, boosting productivity, and promoting sustainability. Maintaining optimal water quality parameters is also essential. Poor water quality can induce stress and suppress the fish's immune system, leading to disease outbreaks. Using inorganic and organic materials to regulate primary production and water quality parameters may have adverse effects on fish health due to residual effects. Ciani et al. show that using calcium oxide (CaO) particles to treat fish farm water can help reduce populations of sea lice, plankton, and echinoderms. The proper dose and frequency of use effectively reduce unwanted biological entities, but CaO exposure did not induce fish mortality or histopathological damage in the skin, eyes, or intestines. This highlights that the proper use of chemicals to maintain water quality parameters is crucial for maintaining the health and welfare of fish.

Genetics is also a very important factor; it has become a key criterion in modern fish health management, enabling the development of disease-resistant strains. Selective breeding enables the production of naturally inherited offspring that are more resistant to pathogens and diseases and that grow faster. Subsequently, dependence on vaccines, functional feeds, and disease-control agents can be reduced, thereby improving both profitability and sustainability of aquaculture. A study published by Preston et al. reports that selective breeding of Atlantic salmon produces individuals that are more resilient to Complex Gill Disease (CGD) and suggests that this could be a practical strategy for mitigating gill damage caused by CGD in farmed Atlantic salmon.

Many fish diseases are reported, some of which are unique to the region or species, but as aquaculture diversifies, these diseases are no longer confined to specific areas or species. Reports indicate that disease transmission occurs frequently between areas or between species. Transmission also occurs from farm to wild or from wild to farm through various means, such as water currents, escaped fish, and equipment. Roh and Kannimuthu developed a predictive model to estimate the prevalence of infectious diseases under different epidemiological circumstances and quarantine measures. Predictive models are important for implementing proper guidelines and SOPs to maintain hygiene and biosecurity, as well as for designing surveillance programs and policies at regional and global scales to prevent the transfer of infectious pathogens.

Some disease diagnostic laboratories have been established as part of a surveillance program to prevent the transmission of diseases from one place to another. However, the uniformity, quality, and efficacy of laboratory diagnostic surveillance (LDS) systems vary across laboratories (Chikh et al.). Therefore, evaluating epidemiological surveillance systems using the OASIS evaluation tool is vital, and implementing these tools enables us to improve disease diagnostic surveillance and frameworks.

Aquaculture is key to meeting global food needs, but infectious diseases pose a major concern for these efforts. Improvements in disease detection, prevention, and monitoring help achieve sustainable aquaculture production. This Research Topic presents a collection of articles offering insights and recommendations for future research in aquatic animal health and epidemiology.

